# Aardvark: sifting through differences in a mound of variants

**DOI:** 10.1186/s13059-026-04165-0

**Published:** 2026-06-18

**Authors:** James M. Holt, Christopher T. Saunders, Egor Dolzhenko, Peter Krusche, Nathan D. Olson, Justin M. Zook, Michael A. Eberle, Zev Kronenberg

**Affiliations:** 1 PacBio, 1305 O’Brien Drive, Menlo Park, 94025 CA USA; 2 Novartis Pharma AG, Basel, CH-4056 Switzerland; 3 Material Measurement Laboratory, National Institute of Standards and Technology, Gaithersburg, 20899 MD USA

**Keywords:** Variant benchmarking, Variant calling, Tandem repeats, Structural variants

## Abstract

Variant benchmarking is critical in assessing the accuracy of genomic secondary pipelines. However, traditional benchmarking tools that require exact genotype matches inject biases from variant representation and are ill-suited for tandem repeat or structural variation. We describe Aardvark, a variant benchmarking tool that introduces the basepair score to directly compare haplotype sequences, reducing representation biases while allowing for partial credit scoring. The tool also includes the traditional genotype score and supports separate or joint benchmarking of small variants, tandem repeats, and structural variants (<10 kb). Aardvark accepts standard inputs, runs ≈18x faster than hap.py, and is open source (https://github.com/PacificBiosciences/aardvark or https://doi.org/10.5281/zenodo.20271610).

**Supplementary Information:**

The online version contains supplementary material available at 10.1186/s13059-026-04165-0.

## Background

Precise genomic variant comparison is fundamental to improving sequencing technologies and ensuring accurate variant calling for research and clinical applications. Therefore, it is critical that variant comparison tools, also known as benchmarking tools, provide the best means to identify real differences between variant call sets by minimizing the representation issues that persist across technology platforms and variant calling software. While sequencing technologies have advanced, there has not been much active development on variant benchmarking tools because they require a great deal of care to properly implement and are often viewed as a resource rather than scientific advancement. As technologies improve, the fraction of false positive/negative variants will shift from true addressable errors to benchmarking issues, slowing the pace of technological improvement. This justifies a renewed effort to improve variant comparison tools, as well as the genomic truthsets they rely on.

The earliest genome-scale benchmarks from Genome in a Bottle (GIAB) focused on non-repetitive genomic regions without structural variation, where variants are relatively easy to consistently detect across a wide range of technologies [1]. However, technical limitations restricted these early benchmarks to primarily unphased small variants, limiting the scope of benchmarking that could be performed. Recent benchmarks have been extended to generate sequence-resolved haplotypes that span more difficult regions of the genome [2–4] by leveraging a mixture of complementary sequencing technologies, advancements in variant calling and *de novo* assembly algorithms, and pedigree inheritance information. For example, the GIAB Telomere-to-Telomere (GIAB-T2T) [3] and Platinum Pedigree (PlatPed) [4] benchmarks include phased small variants, tandem repeats, and structural variants (≥50 bp). The benchmarking truthsets now cover the majority of the human genome as haplotype resolved sequences. For variant benchmarking, these haplotype sequences are reduced to variant calls relative to a reference genome (i.e., VCF files) and associated high-confidence regions.

The most common tool for performing small variant benchmarking is hap.py [5] with vcfeval [6] as the variant comparison engine. Hap.py normalizes variants which reduces variant comparison errors between the truth and query sets, and vcfeval maximizes the number of detected true positives in the dataset through dynamic programming. Ultimately, matching variants between the truth and query sets are labeled as true positives (TP), and the mismatches are labeled as false negatives (FN) or false positives (FP), respectively. Hap.py is a genotype-centric approach, meaning that each variant genotype in the truth and query is assigned a single label, and those labels are used to calculate recall, precision, and F1 scores. There are important limitations and implicit biases in genotype-centric approaches that under-utilize modern haplotype-resolved truth sets.

Genotype-centric benchmarking scores all variants equivalently regardless of size, meaning all indels from 1–50 bp are given the same weight, even though larger indels are more likely to be deleterious [7]. Additionally, equivalent variant representations can be scored differently despite encoding the same underlying genetic change. For instance, in certain genomic contexts, a 2-bp insertion may be represented either as a single 2-bp VCF entry or as two adjacent 1-bp entries. Under genotype-centric scoring, the single 2-bp insertion is counted once, whereas the two 1-bp insertions are counted twice, effectively doubling the weight. This introduces biases for or against particular variant representations and can lead to different performance metrics even when the compared variants produce sequence-identical haplotypes [8]. While these limitations may be acceptable in coding regions where outcomes are expected to be binary (right or wrong), it performs poorly in repeat-rich regions such as tandem repeats where indels are common and highly variable in length, making genotype-centric accuracy an unstable metric for comparative evaluations.

Critically, genotype-centric benchmarking does not allow for partial credit, which is becoming increasingly relevant as the benchmarks shift towards sequence-resolved haplotypes in repetitive regions and with larger forms of variation. Inexact allele matches often occur around larger indels, where precisely identifying the full variant sequence has more potential for error, particularly in homopolymers and tandem repeats. Similarly, tandem repeat variants have historically been analyzed by just looking at the expansion or contraction length, but recent studies show sequence composition is important for tandem repeat analysis [9]. Tools like Truvari [10] use partial credit to benchmark tandem repeats, and distill the comparison to a binary outcome (right or wrong) based on sequence identity. Vcfdist [8, 11] follows similar logic for both small variants and medium-sized structural variants (<10 kb). While both Truvari and vcfdist use partial credit to assist in matching and scoring variants, they are both still genotype-centric approaches, assigning equal weight to each genotype in the file and leaving them subject to similar representation biases as hap.py/vcfeval.

These limitations are alleviated by sequence-based evaluations. In particular, the recent Telomere-to-Telomere “genome benchmark” and accompanying software (“Genome Quality Checker”) compare genome-scale sequences (i.e., assemblies) instead of variants [12]. With a sequence-based approach, the summary metrics are based on the number of true or false basepairs, which is a natural way to analyze sequence differences.

Here we present Aardvark, an open-source benchmarking tool implemented in Rust for small to midsized variants (1 bp–10 kb) that provides both genotype-centric and sequence-centric benchmarking strategies, achieving near parity with hap.py while introducing a basepair-level evaluation framework. Its sequence-centric (“basepair” or “BP”) scoring reconstructs benchmark and query haplotypes and compares them at the basepair level, making it variant-type agnostic, weighting each altered base equally, and reducing representation biases inherent to genotype-based approaches. This makes it well-suited for small variants (SNV/indel), tandem repeats, and medium-sized structural variants (<10 kb). The tool accepts standard formats and produces summary metrics, labeled VCFs, per-region metrics, and stratified results, while running approximately 18x faster than hap.py to support rapid method development.

## Results

### Aardvark for variant benchmarking

**Fig. 1:**
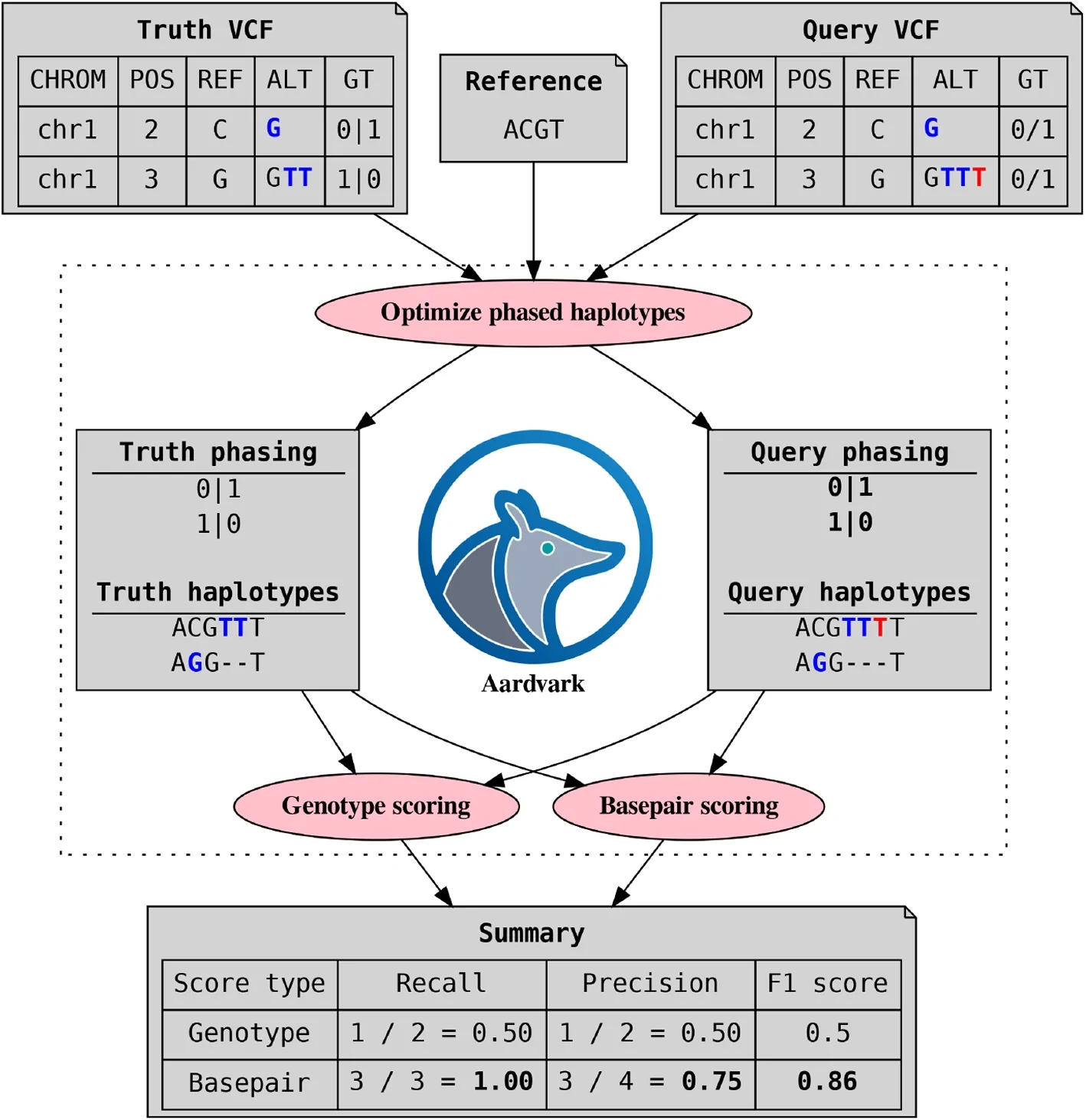
Aardvark process overview. Aardvark ingests a reference genome file (FASTA) and two VCF files: one for truth variants and one for query variants. It then calculates the optimal phasing of the query variants such that the edit distance between the truth and query haplotypes is minimized. The phased variants are then independently scored using the genotype and basepair scoring schemes. Metrics from both scores are provided in a summary file. In this example, the truth and query VCFs share a single-nucleotide variant, but the insertion variant in the query VCF is one basepair longer than the one in the truth VCF. For genotype scoring, this one basepair difference creates both a false positive and false negative for the insertions, leading to recall and precision of 0.500. For basepair scoring, only bases that are different from the reference sequence are scored, ignoring any non-variant bases. We have highlighted the bases that are scored as true positives in blue, and the single extra false positive “T” base in red, leading to more informative recall, precision, and F1 in the basepair score

Aardvark is a variant benchmarking framework that provides a new sequence-centric (“basepair” or “BP”) and a traditional genotype-centric (GT) approach for comparing variants. The tool clusters variants into sub-regions and analyzes them independently, in parallel. For each region, the algorithm searches for an optimal phase orientation of the query heterozygous variants to minimize conflicts with the truth set. Truth set phasing is always respected, whereas phasing in the query set is ignored due to the possibility of phasing errors. The phase-optimized variants are transformed into local haplotype sequences that are then evaluated with both the basepair and genotype scoring schemes (see Fig. 1). Aardvark’s genotype score was designed to provide near-parity with the *de facto* benchmarking tool (hap.py, [5]) while improving usability, portability, and runtime. Throughout our results, we demonstrate the utility of the new basepair comparison mode.

### Aardvark generates sequence-centric summary metrics

We started our analysis by systematically characterizing the differences between basepair and genotype scoring under controlled conditions using minimal synthetic examples. Four distinct scenarios were constructed (Table 1): heterozygous single-base variants, homozygous variants, heterozygous insertions, and a complex mixture of variant types. These examples demonstrate that basepair scoring assigns weight proportional to variant length, resulting in higher weights for longer variants. We then examined how representation biases and zygosity influence genotype and basepair scoring (Additional file 1: Table S6). The total weight of the genotype score is fixed by the number of variant records in the VCF file, regardless of zygosity, whereas the basepair score is weighted according to the total number of altered basepairs. We provide a common example where two variant sets (single SNV v. two indels) generate the same exact haplotype but are scored differently with genotype scoring (Additional file 1: Fig. S9). Lastly, we outline how basepair scoring addresses critiques of genotype scoring (Additional file 1: Table S4). These examples highlight the nuance of comparing even simple genomic variation, and the importance of considering sequence length impact (with BP), rather than just counting variants (with GT).

**Table 1:** Examples of differences between Aardvark’s genotype (GT) and basepair (BP) scoring schemes. The examples in this table include the truth sequence, the query sequence, and the generated number of true positives (TP), false negatives (FN), and false positives (FP) for both genotype and basepair scoring schemes. For all examples, the reference allele is “A”. To avoid floating-point numbers, BP values sum to twice the number of altered basepairs. Set A are all heterozygous variants with a single base change, including two examples where the incorrect alternate allele is detected. Set B includes at least one homozygous variant in each set, showing how BP separately scores each allele. Set C are all heterozygous insertions, showing major scoring differences due to implicit partial credit. Set D includes a mix of heterozygous and homozygous genotype calls on alleles that reflect common errors in homopolymer variant calling. For genotype scoring, TP may be different between truth and query (impacting recall and precision) and is annotated as “Truth.TP” and “Query.TP” where relevant. If TP, FN, or FP is unlisted, it is =0

Set	Truth	Query	GT	BP
A	A/C	A/C	TP=1	TP=2
	A/C	A/A	FN=1	FN=2
	A/A	A/C	FP=1	FP=2
	A/C	A/G	FN=1, FP=1	TP=1, FN=1, FP=1
	A/C	A/AC	FN=1, FP=1	TP=1, FN=1, FP=1
B	C/C	C/C	TP=1	TP=4
	C/C	A/C	FN=1, Query.TP=1	TP=2, FN=2
	C/C	A/A	FN=1	FN=4
	A/C	C/C	Truth.TP=1, FP=1	TP=2, FP=2
	A/A	C/C	FP=1	FP=4
C	A/ACCC	A/ACCC	TP=1	TP=6
	A/ACCC	A/ACC	FN=1, FP=1	TP=4, FN=2
	A/ACCC	A/ACCCC	FN=1, FP=1	TP=6, FP=2
D	C/C	C/AC	FN=1, Query.TP=1, FP=1	TP=3, FN=1, FP=1
	ACCC/ACCC	ACC/ACCCC	FN=1, FP=1	TP=10, FN=2, FP=2

### Basepair scoring emphasizes sequence correctness

**Fig. 2:**
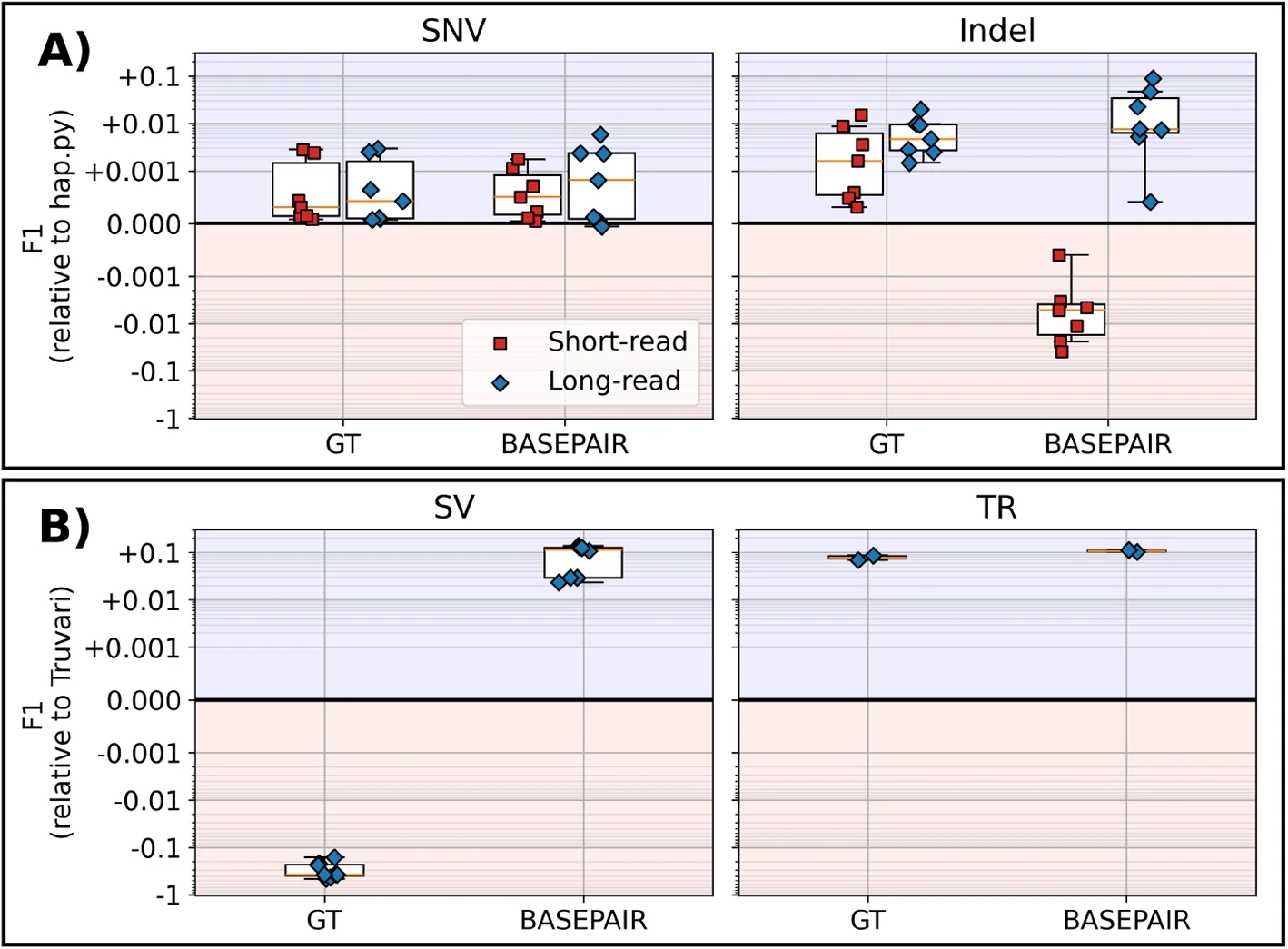
Aardvark F1 accuracy relative to other common variant comparison tools. **A** Small variant F1 scores for 14 Aardvark benchmarks relative to hap.py for single nucleotide variants (SNV, left) and indels (right). The genotyping scoring scheme is nearly identical to hap.py, with slightly increased F1 scores driven by minor differences in counting zygosity errors and improvements in representation matching. Basepair scoring considers indel length, resulting in lower F1 scores for the short-read query sets and higher scores for long-read query sets. **B** Large variant F1 scores for 8 Aardvark benchmarks relative to Truvari, including structural variants (SV, left) and tandem repeat variants (TR, right). Unlike Truvari, Aardvark SV genotype scoring does not allow for “fuzzy” matching resulting in lower GT scores, whereas basepair provides partial credit based on edit distances. For tandem repeats, Aardvark shows nearly a 10% increase in F1, as basepair scoring is a more natural framework for difficult repeat variants

To understand the behavior of basepair scoring relative to genotype scoring with real-world data, we ran hap.py [5] and Aardvark, and then compared their F1 scores (the harmonic mean of recall and precision) while stratifying by variant type. We tested multiple combinations of publicly available truth benchmarks [1–4] in conjunction with query datasets [13, 14] generated from a diverse set of sequencing technologies and variant calling pipelines (Additional file 1: Tables S1 and S2). For SNVs, we see a near parity between basepair scoring and hap.py (mean F1 delta: +0.0010, Fig. 2A), which is expected given that SNVs implicitly have a weight of 1 bp. For indels, the basepair F1 score relative to hap.py is lower for short-read query sets (mean delta: -0.0126) but higher for long-read query sets (mean delta: +0.0255). We found that the short-read query sets excel at shorter indels (≤15 bp) but struggle with longer indels (>15 bp, Additional file 1: Fig. S6), a pattern that persisted with datasets from the Precision FDA Truth Challenge v2 ([15], Additional file 1: Fig. S7). These longer indels alter more sequence and therefore have increased relative weight with basepair scoring. This culminates in basepair F1 scores that are lower than the genotype F1 scores for these short-read query sets. In contrast, the long-read query sets had better performance with the longer indels, leading to increased F1 scores even when the reported alleles included minor sequence errors.

**Fig. 3:**
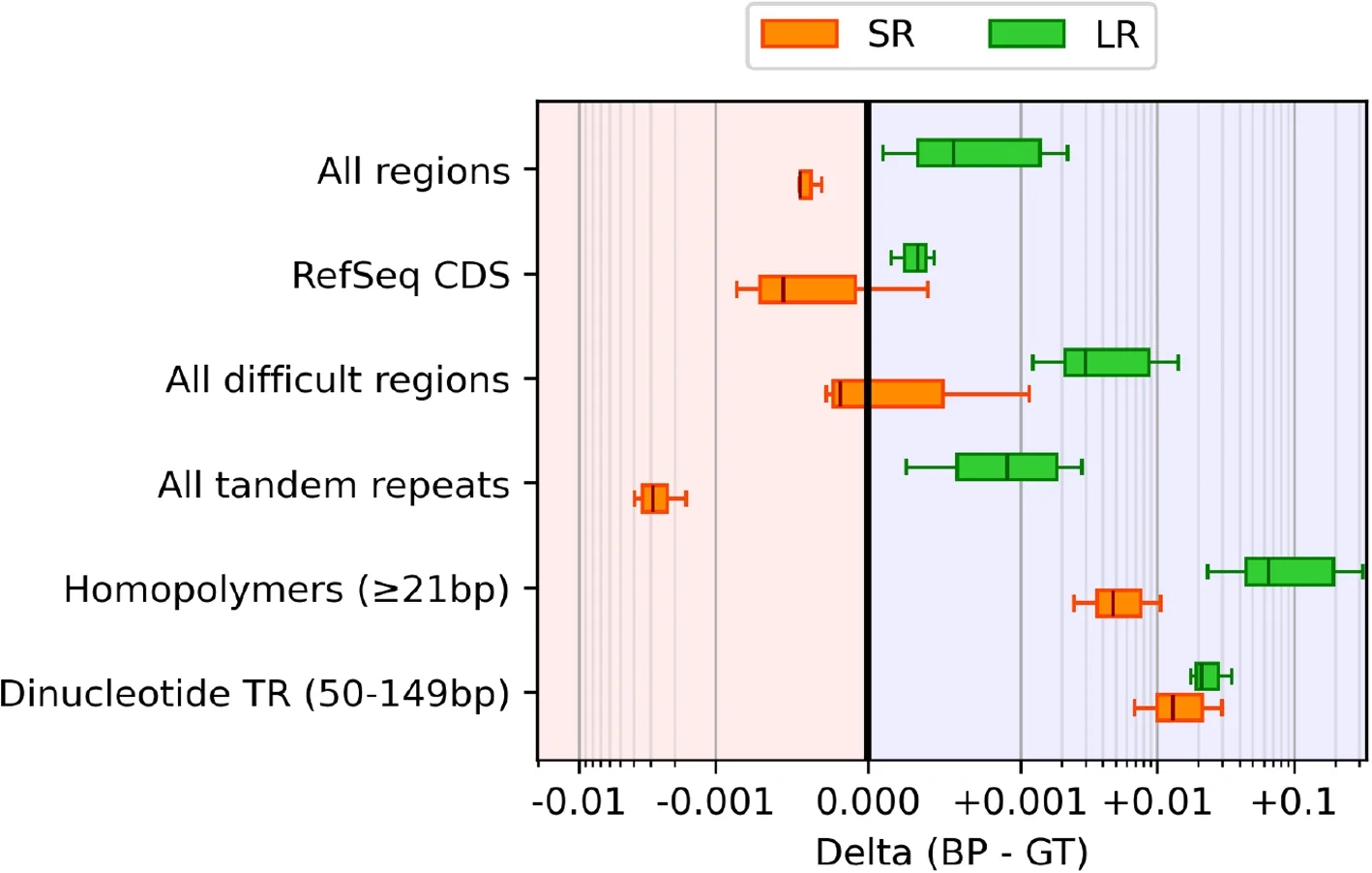
Stratification of Aardvark-GT and -BP by genomic context. This figure shows the difference between genotype (GT) and basepair (BP) scoring for both short-read (SR) and long-read (LR) query sets across multiple benchmark comparisons. On average, the basepair F1 scores are lower for short-read query sets and higher for long-read query sets for “All regions” and the RefSeq coding regions (“RefSeq CDS”). However, all technologies showed improved basepair scores in homopolymer and dinucleotide tandem repeats (TR), highlighting that exact-matching scoring (GT) often under-represents the sequence-level accuracy in low-complexity repeat regions

We sought to identify which genomic contexts are most affected by shifting from genotype to basepair scoring. Using standard genome annotation sets (e.g., repeats, difficult regions, and coding regions), we stratified F1 scores for both short-read and long-read query sets (Fig. 3). In tandem repeat regions, the basepair scores were lower for short-read query sets (mean delta: -0.0028) but higher for long-read query sets (mean delta: +0.0013). Short-read query sets exhibit reduced F1 scores in these regions due to decreased accuracy of larger indels. Although such variants are relatively infrequent by count, they account for a disproportionate share of altered basepairs, and therefore contribute more strongly to BP-based performance metrics than to GT-based metrics. However, when further restricted to homopolymer or dinucleotide repeats, the basepair F1 score is consistently higher than genotype F1 score across all tested technologies (mean deltas: +0.0714 and +0.0207, respectively). In these regions, variant callers frequently produce inexact variant alleles due to common sequencing errors. Basepair scoring detects these partial matches and assigns credit proportional to the correctness of the allele, instead of marking the entire variant as false. Based on these regional stratifications, we conclude that basepair scoring is particularly well-suited to divergent genomic regions that are enriched for indels and other low-complexity variants.

### Aardvark benchmarks tandem repeat and structural variants

Aardvark’s basepair scoring is well-suited for benchmarking variants up to 10 kbp in length, which includes the majority of tandem repeat (TR) and structural variants (SV). Larger variants (>10 kbp) are prohibitively expensive to analyze in our current framework and are often not sequence-resolved, which is a requirement for basepair scoring. Both SV and TR variant calls have some of the highest allelic representation differences, with varied positions, lengths, and sequence content. We used both Aardvark-GT and -BP to compare multiple structural or tandem repeat variant query sets from long-read sequencing callers (pbsv, sawfish [16], and TRGT [17]) against the Platinum Pedigree [4] and GIAB Telomere-to-Telomere [3] benchmarks. We evaluated each combination using both Truvari [10] and Aardvark (Fig. 2B). For SVs across all benchmark comparisons, Aardvark-GT is significantly lower than Truvari (mean delta: -0.3315), highlighting the importance of the inexact (or “fuzzy”) matching implemented in Truvari for comparing SVs with a genotype-centric approach. However, Aardvark’s basepair score is higher than Truvari (mean delta: +0.0875). Similarly, the basepair score for TRs is also higher than Truvari (mean delta: +0.1083), indicating that basepair scoring better captures the sequence-level correctness of these larger forms of variation, and that our variant calling tools are correctly identifying more altered basepairs than the Truvari scores imply.

### Basepair scoring reduces representation biases in tandem repeats

Tandem repeat regions contain many small indels [18] where representation issues can accumulate. In theory, Aardvark’s basepair scoring should alleviate these issues by faithfully recapitulating and comparing the underlying haplotypes. To test this hypothesis, we carried out a dual-truthset benchmarking experiment, comparing SNV/indel (DeepVariant, [19]) calls against both the small variant and the tandem repeat benchmarks of the Platinum Pedigree [4]. Similarly, we compared tandem repeat calls (TRGT, [17]) to both benchmarks. There were ≈ 413 K regions spanning ≈41 Mbp where the underlying haplotype sequences for the small variant and tandem repeat truth sets perfectly agreed (Additional file 1: Section S3.5). In these regions, we would expect identical benchmarking outcomes when compared to the same query set. The results of our experiment (Table 2) demonstrate that Aardvark’s genotype score wobbles (±0.0025) when these benchmarks are swapped, indicating that GT scores are sensitive to representation even within these interchangeable regions. In contrast, Aardvark’s basepair scores remained identical across benchmarks, as basepair scoring is insensitive to representation issues. Additionally, the basepair F1 scores are consistently higher than the corresponding genotype F1 scores, highlighting the importance of sequence-level comparisons with partial credit for quantifying variation in tandem repeat regions. This increase is most prominent with the tandem repeat query set (+0.0164), where any sequence-level mismatch forces the entire variant to be labeled false under genotype-based scoring. Therefore, we posit that Aardvark’s basepair scoring scheme is the preferred approach for quantifying tandem repeat calling accuracy.

**Table 2:** Aardvark comparisons in Platinum Pedigree tandem repeat regions. This table shows the F1 score from Aardvark for different combinations of truth and query sets restricted to tandem repeat regions in the Platinum Pedigree benchmarks. The genotype F1 scores (“GT F1”) show variability in the results due to representation biases and binary pass/fail scoring. In contrast, the basepair F1 scores (“BP F1”) are identical for a query set regardless of the comparator truth set, and they are consistently higher than the genotype F1 scores. In summary, this highlights that exact-match genotype scoring is susceptible to representation bias and under-represents the sequence-level accuracy in tandem repeat regions

Query set caller	Truth set	GT F1	BP F1 (Delta)
DeepVariant	Small variants	0.9853	0.9923 (+0.0070)
	Tandem repeats	0.9828	0.9923 (+0.0095)
TRGT	Small variants	0.9786	0.9949 (+0.0163)
	Tandem repeats	0.9785	0.9949 (+0.0164)

### Aardvark for joint benchmarking of different variant classes

One feature that is unique to Aardvark is the ability to benchmark integrated call sets composed of small (SNV/indels) and large variants (TR/SVs) with both genotype and basepair scoring. We demonstrate this feature by benchmarking against the full GIAB Telomere-to-Telomere benchmark [3]. For a query set comparator, we ran an experimental joint variant calling pipeline (longcallD, [20]) that produces integrated small and structural variants in a single VCF file. While the structural variants make up only 1.32% of the joint benchmark genotypes, they cover 65.06% of the altered basepairs in the truth set, which translates into a roughly 3-fold increase in the total number of altered basepairs relative to the small variants alone. With the joint comparison, Aardvark calculates a structural variant genotype F1 score of 0.8740 and a basepair F1 score of 0.9130. These values are lower than the small variant F1 scores, leading to reduced combined F1 scores when considering all variants together (Table 3). The impact on the overall genotype F1 score is low (-0.0048) because the number of structural variants in the dataset is a relatively small fraction of the total variants (1.32%). While there are few structural variants by count, they impact significantly more basepairs than the small variants, which translates to more weight (65.06%) with basepair scoring and a larger drop in the basepair F1 score (-0.0438) when all variants are considered together.

We found that increasing the size of Aardvark’s sub-problems (genomic regions) led to improved accuracy scores for the full benchmark comparison. This is controlled by the “window size” parameter, which sets how close two variants need to be to go into the same sub-problem. By increasing the window size from the default of 50 bp up to 1 kb, we increase the overall basepair F1 score by +0.0110, but at the cost of increased compute time (from ≈3 minutes to ≈10 minutes with 16 threads). We do not observe this F1 score improvement with only small variant comparisons (Additional file 1: Fig. S8), suggesting that the difference is caused by the inclusion of structural variants and/or the more challenging regions containing them.

**Table 3:** Example joint Aardvark comparisons. This table shows multiple Aardvark comparisons of the GIAB-T2T benchmark against a query set generated from an experimental joint variant calling pipeline based on *de novo* assembly. Both the truth and query sets include integrated small and structural variants in a single VCF file. The first comparison is limited to the GIAB-T2T small variant regions, whereas the second and third comparisons are against the full GIAB-T2T benchmark which includes more challenging regions with structural variants. For both GIAB-T2T region sets, we show the percentage of benchmark variation that is structural (“Percent SV”) from both genotype (GT) and basepair (BP) perspectives. The first two comparisons used Aardvark’s default window size of 50 bp, and we include a third comparison that increases it to 1 kb. The F1 scores from both genotype and basepair scoring is shown for all variants and structural variants alone (“SVs F1”)

T2T regions	Percent SV		Window	All variants F1		SVs F1	
	GT	BP	Size	GT	BP	GT	BP
Small variants	–	–	50	0.9842	0.9874	–	–
Full benchmark	1.32%	65.06%	50	0.9794	0.9436	0.8740	0.9130
			1000	0.9802	0.9546	0.8964	0.9181

## Discussion

Aardvark’s basepair score provides a sequence-level comparison that addresses many of the critiques of genotype scoring. Specifically, basepair scoring 1) reduces or removes biases from variant representation, 2) equalizes the weight of each altered basepair, and 3) enables partial credit when allelic sequences do not exactly match. Basepair scoring is analogous to the “genome benchmark” approach [12], but it operates on smaller local haplotypes derived from VCF files and does not require fully-phased variant calls for the benchmark and query sets. The genotype score is intended to serve as a replacement for hap.py with practical enhancements in the form of improved speed and greater ability to resolve complex variation. We consider these two scoring schemes to be complementary, with basepair scoring providing an approach that is much closer to complete haplotype-resolved benchmarking, whereas genotype scoring may be more suited for applications where strict exact-matching variants are critically important such as evaluation of variant calls in clinically-relevant regions.

Aardvark’s basepair scoring scheme provides a benchmarking view that is useful for improving variant callers. First, the basepair approach enables comparisons that were previously challenging or heavily biased due to representation issues. For example, we demonstrated how Platinum Pedigree tandem repeats and small variants benchmarks can be used interchangeably to achieve the same basepair score metrics, enabling developers to perform benchmarks that were previously inaccessible due to significant differences in variant representation. Second, the increased resolution from basepair scoring enables stratification of regions by their degree of haplotype correctness, instead of a binary pass/fail score. This can help developers categorize errors by sequence composition and/or severity, enabling more focused assessment and development of tools. Lastly, variant callers that leverage machine learning [13, 19, 21] could be modified to use the basepair scoring metrics as part of the optimization error to minimize. A genotype-based score may get caught in local minima where improvements to imperfect allelic sequences may manifest as identical scores. Basepair scoring could allow the training to escape these local minima through the detection of partially correct results, which may lead to faster convergence or increased accuracy in the final variant calling models. In other words, for variants that are difficult to call, basepair scoring rewards incremental improvements towards the correct sequence.

We demonstrated Aardvark’s ability to jointly benchmark different classes of variation, but there are not many available resources or variant callers that can fully leverage this capability. To our knowledge, the GIAB-T2T benchmark is the only available truth set with integrated small and structural variant calls in a single file. All other benchmarks in our experiments split the variants into different files, allowing for focused comparisons of a class of variation but at the cost of potentially duplicate or conflicting variants. Similarly, variant callers (i.e., query sets) are often specialized to a class of variation, which can lead to conflicting and overlapping variant calls that require additional computation to resolve. Recent assembly-based variant callers will report all variant types in one phased VCF without conflicts [22, 23], but these callers are inherently limited by the accuracy and completeness of the underlying assembly. While comprehensive joint benchmark resources and variant callers may not be quite ready for routine use, we hope to leverage the capabilities of Aardvark to benchmark their outputs and identify areas for future improvement.

## Conclusions

Aardvark is a new variant comparison and benchmarking tool that introduces the basepair scoring scheme, enabling direct haplotype sequence comparisons and reducing biases from variant representation. Additionally, Aardvark supports joint benchmarking of small variants, tandem repeats, and structural variants, enabling more comprehensive comparisons with assembly-based benchmarks and variant callers. The implementation is computationally efficient while reporting both the basepair score and a traditional genotype score. The tool ingests standard file formats and outputs labeled variants, summary statistics, and several optional files for users who desire deeper details on each individual haplotype comparison. Aardvark is open source, easy to install, and freely available on GitHub (https://github.com/PacificBiosciences/aardvark or https://doi.org/10.5281/zenodo.20271610).

## Methods

### Overview

Aardvark prioritizes a sequence-centric approach to variant benchmarking. Thus, the core algorithmic problem is to efficiently compare diploid haplotype sequences that are derived from two sets of input variants: “truth” and “query” sets. To take advantage of parallelism, Aardvark first divides the problem space into sub-regions that are solved independently. For each sub-region, Aardvark will 1) construct optimized truth and query haplotype sequence pairs from the provided variants such that the total edit distance between the pairs is minimized, and 2) score the optimization solution using multiple scoring schemes.

### Region identification

Performing a full end-to-end comparison for an entire chromosome is computationally challenging. Additionally, truth sets often come with “confidence” or “benchmark” regions indicating where the comparison should be performed for the highest accuracies, effectively excluding regions where the truth sets do not contain high-confidence variation. Aardvark starts with these confidence regions, and further divides them into sub-regions based on the input variants and a user-defined window heuristic (default: W=50 bp). When parsing the truth and query variants, any variants from either input that are within ±W basepairs of each other are grouped together into a sub-problem. These sub-problems are analyzed independently, allowing for Aardvark to leverage parallelization by distributing the sub-problems to different threads. This windowing scheme is similar to approaches taken by other tools to sub-divide the problem [5, 8]. Increasing the window size creates larger sub-problems and may improve accuracy under certain conditions such as when larger variants are part of the benchmark, but it also increases the computational complexity of each sub-problem. When restricted to only small variants, we found negligible differences in the summary F1 scores beyond the default 50 bp window size, and the additional compute requirements started to become noticeable beyond 200 bp (Additional file 1: Fig. S8). For structural and tandem repeat variants, we found 1 kb to be a suitable window size for most applications. While this heuristic window size approach is computationally fast, more sophisticated strategies like those of vcfdist [11] may prove to be better for accuracy.

### Sequence optimization

For each sub-problem, Aardvark starts by constructing two pairs of truth and query haplotype sequences (four total) that minimize the total edit distance between each truth/query sequence pair. These sequences are constructed using the variants and zygosities provided in the truth and query variant files. A homozygous variant must be incorporated into both corresponding haplotype sequences (e.g., a truth variant must go into both truth1 and truth2). However, a heterozygous variant is incorporated into exactly one of the two haplotypes, and the phase orientation defines which haplotype incorporates the variant (e.g., a truth variant with orientation 1|0 will be incorporated into truth1, but not truth2). Thus, the core problem is to find an optimal combination of *phased* zygosities such that the generated haplotype sequences have the smallest combined edit distance (e.g., minimize ed(truth1, query1) + ed(truth2, query2)).

When phased genotypes are provided for the truth variants, Aardvark will respect them, meaning that the algorithm will not alter or “flip” the phase orientation of any phased truth zygosities. If the truth variants are unphased, the algorithm is allowed to flip their phase orientations to minimize the edit distance calculations. In contrast, query phase orientations are never respected as it is assumed that phasing errors are possible in the provided data. For most modern truth sets, the variants are all phased onto maternal and paternal haplotypes, so this routine is typically only searching for the optimal query phase orientations.

Given *V* total unphased heterozygous variants, there are $2^V$ possible phase orientations that exist. Aardvark models this search space as a decision tree where at depth *V* the phase of the *V*th variant (ordered by position) is assigned. Aardvark explores this decision tree using a lowest-cost-first algorithm based on the total edit distance between the truth and query haplotype sequences represented by the node. The method is initialized at the root node (V=0) with no assigned zygosities. Each possible phase orientation is explored and added to the minimum cost queue using the edit distances derived from comparing the partial haplotypes. This process is repeated until depth *V* is reached, indicating that all variants have been phased and a minimum-cost solution has been found. Conceptually, this process is similar to the phasing algorithm described for HiPhase [24], but with a slightly altered search space and optimization scheme adapted for this problem.

### Edit distance calculations

The underlying edit distance calculations are handled by a custom dynamic version of the wavefront algorithm [25] (WFA). The base WFA algorithm is very efficient, with run-time scaling based on the edit distance between two sequences. Aardvark’s dynamic version allows for the pair of sequences (truth and query) to each be extended as the search algorithm traverses down the decision tree. Additionally, this method does not apply a penalty if there are extra characters in the partially constructed sequences, as future extensions may lead to matches. Given that the vast majority of haplotypes are expected to exactly match for high-quality variant calling pipelines, this method of exploring the search space often traverses directly down the search tree to the correct answer in *O*(*V*) time, with negligible computation cost from the WFA calculations. However, regions with higher error rates can lead to much more branching ($O(2^V)$) with higher WFA costs, thus Aardvark bounds the search by limiting the number of nodes searched at each depth to a fixed heuristic value (*H*), limiting the worst case search to *O*(*HV*). In practice, we found that Aardvark rarely hits these limits, but when it does it is usually in areas that are enriched for false positives and/or negatives (i.e., the truth/query haplotypes have significant divergence).

### Scoring

After identifying the optimal phase orientation for all truth and query variants, Aardvark scores each pair of haplotypes independently (truth v. query) and combines the scores for the region. Aardvark scores the differences at the sequence (or haplotype) level, fully removing variant representation from consideration for reporting the results in the basepair (BASEPAIR) scoring scheme. The tool also reports genotype (GT) level metrics which are highly similar in both method and outcome to those reported by previous methods [5, 6]. Aardvark also reports two other genotype-based scores with alternate weights (Additional file 1: Section S3.2).

#### Basepair scoring scheme

Aardvark’s basepair scoring scheme directly compares two haplotypes sequences without knowledge of the underlying variants. This approach starts by constructing haplotype sequences from the optimized truth and query variant phasing, effectively blinding all downstream computations to the original variant representations. For both pairs of truth/query haplotypes, it counts the number of bases in the haplotype sequences that are true positives (changes shared in both truth and query relative to the reference sequence), false negatives (changes unique to truth), or false positives (changes unique to query). Thus, the denominator in metrics like precision and recall scaled with the total number of changed, inserted, or deleted basepairs relative to the reference sequence.

By definition, all differences between the reference genome sequence and a truth haplotype sequence must be either a true positive or a false negative. Similarly, each difference between the reference sequence and a query haplotype sequence must be either a true positive or a false positive. Lastly, each difference between the truth and query haplotypes must be either a false negative or false positive. Given a method to calculate the edit distance between two sequences, these definitions form a system of equations that can be trivially solved for each pair of haplotype sequences. Let *R* be the reference genome sequence in the region, *T* be the constructed truth haplotype sequence, and *Q* be the constructed query haplotype sequence. Let the edit distances be calculated by some function, *ed*(*A*, *B*), between each pair of input sequences: X=ed(R,T), Y=ed(R,Q), and Z=ed(T,Q). Finally, let *TP* be the number of true positives, let *FN* be the number of false negatives, and let *FP* be the number of false positives. Given these definitions, we create the following system of equations where *TP*, *FN*, and *FP* are initially unknown, and all the edit distances are calculated from the optimized haplotype sequences:

1 $$\begin{aligned} \left\{ \begin{array}{l} TP+FN = ed(R, T) = X\\ TP+FP = ed(R, Q) = Y\\ FN+FP = ed(T, Q) = Z \end{array}\right. \end{aligned}$$

Which simplifies to:

2 $$\begin{aligned} \left\{ \begin{array}{l} TP = \frac{X+Y-Z}{2} \\ FN = X - TP \\ FP = Y - TP \end{array}\right. \end{aligned}$$

This system of equations is solved twice, once for each pair of optimized truth and query haplotype sequences. Interestingly, these definitions also allow for TP, FN, and FP values that are half-correct (i.e., include a 0.5 fractional component). These half-correct bases typically represent situations where the truth and query both incorporate alternate alleles, but with slightly different sequences. In practice, Aardvark doubles all basepair scoring counts (TP, FN, and FP) to remove floating-point calculations as a source of technical error. Table 1 includes several examples demonstrating the doubled base counts and partially correct sequences.

The core benefit of the basepair scoring scheme is that variant representation biases have been removed entirely. First, the total weight of any comparison is always equal to the number of altered basepairs, removing biases from zygosity or variant representation. Second, equivalent variant representations have identical summary recall values when errors are present (Additional file 1: Table S6). This enables greater resolution in the summary metrics while also significantly reducing biases that may be injected from alternate variant representations.

#### Genotype scoring scheme

For the genotype scoring scheme, Aardvark starts with the same phase orientations from sequence optimization, but uses an orthogonal algorithm to compute the metrics. For each pair of alternate alleles, Aardvark searches for the maximum number of alternate alleles that can be incorporated into the reference sequence such that the output truth and query haplotype sequences exactly match. Critically, the allele representations do not have to match, so long as the final haplotype sequences are basepair-identical. Any alleles that are incorporated are marked as true positives, and all others are either false negatives or false positives depending on the source. For homozygous genotypes, the genotype score further requires that both alleles are marked as true positives for the entire genotype to also get marked as a true positive. In general, this method is consistent with previous approaches and the outcomes are highly concordant with hap.py (Additional file 1: Fig. S3).

There are two subtleties worth mentioning about the Aardvark implementation of the genotype score. First, any multi-allelic heterozygous sites are split into two distinct heterozygous genotypes for all analyses. Second, if the difference between truth and query is limited to the zygosity of the genotype call (i.e., heterozygous v. homozygous), then the heterozygous call is labeled as “true” and the homozygous call is labeled “false”. For example, if the truth set has a heterozygous call while the query set has homozygous, then the truth variant is labeled as a true positive since it was correctly detected in the query, but the query variant is labeled as a false positive since an extra allele is present. Both of these subtleties are distinctions from the hap.py genotype scoring [5], and are sources of technical discrepancy when comparing the two tools (Additional file 1: Section S2.3).

### Truth and query datasets

Throughout this manuscript, we used a variety of publicly available benchmarks (truth sets), all of which are for the well-studied HG001 (NA12878) or HG002 samples. For HG001 benchmarks, we used Genome in a Bottle (GIAB) v4.2.1 [1] and Platinum Pedigree (PlatPed) v1.2 [4]. For HG002 benchmarks, we used Genome in a Bottle (GIAB) v4.2.1 [1], GIAB Challenging Medically Relevant Genes (GIAB-CMRG) v1.00 [2], and GIAB Telomere-to-Telomere (GIAB-T2T) Q100 v1.1 [3]. The GIAB v4.2.1 sets are relatively strict and focus mostly on non-repetitive genomic regions, meaning they are typically “easier” to resolve correctly with modern sequencing technologies and variant callers. In contrast, the CMRG, PlatPed, and GIAB-T2T benchmarks all include more difficult regions of the genome, which are typically much harder for modern pipelines to resolve correctly.

For query sets, we leveraged a combination of short-read and long-read datasets from previous publications [13, 14] and other public resources. These query sets are generated using a variety of pipelines and sequencing technologies. While efforts were made to select high quality variant calls for these comparisons, this is a rapidly developing field and the approaches used to generate these variant calls may be outdated for a variety of reasons. While the Aardvark tool is intended to analyze the accuracy of variant calling, the analyses provided in this document were chosen to demonstrate the capabilities of the tool and the effect of alternate scoring schemes in practice, and they should *not* be used to compare the underlying technologies or pipelines. As such, each query set is either completely anonymized or labeled with the corresponding read-length type (short-read or long-read), as this was the only relevant feature we identified for understanding the differences between Aardvark’s scoring schemes. Links to the exact files for our benchmark and de-anonymized query sets are available in our Additional file 1: Section S1.1.

## Supplementary Information

Additional file 1. A supplementary PDF file is included with this manuscript that provides details on all used data collections, additional analyses of Aardvark results, and additional details on Aardvark methodology.

## Data Availability

The source code for Aardvark is available under the MIT License on GitHub at https://github.com/PacificBiosciences/aardvark. The source code for the specific version used throughout this manuscript is also on Zenodo [26]. The exact VCF files used in our analyses and the binaries for both Aardvark and Hap.py are available on Zenodo [27] and described in our Additional file 1: Section S1. Web-viewable synthetic examples are available on GitHub at https://github.com/holtjma/mini_variant_benchmarks.

## References

[1] Zook JM, McDaniel J, Olson ND, Wagner J, Parikh H, Heaton H, et al. An open resource for accurately benchmarking small variant and reference calls. Nat Biotechnol. 2019;37(5):561–6. 30936564 10.1038/s41587-019-0074-6 PMC6500473

[2] Wagner J, Olson ND, Harris L, McDaniel J, Cheng H, Fungtammasan A, et al. Curated variation benchmarks for challenging medically relevant autosomal genes. Nat Biotechnol. 2022;40(5):672–80. 35132260 10.1038/s41587-021-01158-1 PMC9117392

[3] Nurk S, Koren S, Rhie A, Rautiainen M, Bzikadze AV, Mikheenko A, et al. The complete sequence of a human genome. Science. 2022;376(6588):44–53. 35357919 10.1126/science.abj6987 PMC9186530

[4] Kronenberg Z, Nolan C, Porubsky D, Mokveld T, Rowell WJ, Lee S, et al. The Platinum Pedigree: a long-read benchmark for genetic variants. Nat Methods. 2025;22(8):1669–76. Nature Publishing Group US New York. 10.1038/s41592-025-02750-y PMC12533576 40759746

[5] Krusche P, Trigg L, Boutros PC, Mason CE, De La Vega FM, Moore BL, et al. Best practices for benchmarking germline small-variant calls in human genomes. Nat Biotechnol. 2019;37(5):555–60. 30858580 10.1038/s41587-019-0054-x PMC6699627

[6] Cleary JG, Braithwaite R, Gaastra K, Hilbush BS, Inglis S, Irvine SA, et al. Comparing variant call files for performance benchmarking of next-generation sequencing variant calling pipelines. BioRxiv. 2015:023754.

[7] Eichler EE. Genetic variation, comparative genomics, and the diagnosis of disease. N Engl J Med. 2019;381(1):64–74. 31269367 10.1056/NEJMra1809315 PMC6681822

[8] Dunn T, Narayanasamy S. vcfdist: Accurately benchmarking phased small variant calls in human genomes. Nat Commun. 2023;14(1):8149. 38071244 10.1038/s41467-023-43876-x PMC10710436

[9] Rajan-Babu IS, Dolzhenko E, Eberle MA, Friedman JM. Sequence composition changes in short tandem repeats: heterogeneity, detection, mechanisms and clinical implications. Nat Rev Genet. 2024;25(7):476–99. 38467784 10.1038/s41576-024-00696-z

[10] English AC, Menon VK, Gibbs RA, Metcalf GA, Sedlazeck FJ. Truvari: refined structural variant comparison preserves allelic diversity. Genome Biol. 2022;23(1):271. 36575487 10.1186/s13059-022-02840-6 PMC9793516

[11] Dunn T, Zook JM, Holt JM, Narayanasamy S. Jointly benchmarking small and structural variant calls with vcfdist. Genome Biol. 2024;25(1):253. 39358801 10.1186/s13059-024-03394-5 PMC11446017

[12] Hansen NF, Dwarshuis N, Ji HJ, Rhie A, Loucks H, Logsdon GA, et al. A complete diploid human genome benchmark for personalized genomics. bioRxiv. 2025:2025–09. 10.1016/j.cell.2026.06.016 PMC13456413 42561913

[13] Behera S, Catreux S, Rossi M, Truong S, Huang Z, Ruehle M, et al. Comprehensive genome analysis and variant detection at scale using DRAGEN. Nat Biotechnol. 2025;43(7):1177–91. Nature Publishing Group US New York. 10.1038/s41587-024-02382-1 PMC12022141 39455800

[14] Porubsky D, Dashnow H, Sasani TA, Logsdon GA, Hallast P, Noyes MD, et al. Human de novo mutation rates from a four-generation pedigree reference. Nature. 2025;643(8071):427–36. Nature Publishing Group UK London. 10.1038/s41586-025-08922-2 PMC12240836 40269156

[15] Olson ND, Wagner J, McDaniel J, Stephens SH, Westreich ST, Prasanna AG, et al. PrecisionFDA Truth Challenge V2: Calling variants from short and long reads in difficult-to-map regions. Cell Genomics. 2022;2(5). 10.1016/j.xgen.2022.100129 PMC9205427 35720974

[16] Saunders CT, Holt JM, Baker DN, Lake JA, Belyeu JR, Kronenberg Z, et al. Sawfish: Improving long-read structural variant discovery and genotyping with local haplotype modeling. Bioinformatics. 2025;41(4):btaf136. 10.1093/bioinformatics/btaf136 PMC12000528 40203061

[17] Dolzhenko E, English A, Dashnow H, De Sena BG, Mokveld T, Rowell WJ, et al. Characterization and visualization of tandem repeats at genome scale. Nat Biotechnol. 2024;42(10):1606–14. 38168995 10.1038/s41587-023-02057-3 PMC11921810

[18] Hannan AJ. Tandem repeats mediating genetic plasticity in health and disease. Nat Rev Genet. 2018;19(5):286–98. 29398703 10.1038/nrg.2017.115

[19] Poplin R, Chang PC, Alexander D, Schwartz S, Colthurst T, Ku A, et al. A universal SNP and small-indel variant caller using deep neural networks. Nat Biotechnol. 2018;36(10):983–7. 30247488 10.1038/nbt.4235

[20] Gao Y, Liao WW, Qin Q, Hall IM, Li H. LongcallD: joint calling and phasing of small, structural and mosaic variants from long reads. bioRxiv. 2026:2026–03.

[21] Zheng Z, Li S, Su J, Leung AWS, Lam TW, Luo R. Symphonizing pileup and full-alignment for deep learning-based long-read variant calling. Nat Comput Sci. 2022;2(12):797–803. 10.1038/s43588-022-00387-x 38177392

[22] Li H, Bloom JM, Farjoun Y, Fleharty M, Gauthier L, Neale B, et al. A synthetic-diploid benchmark for accurate variant-calling evaluation. Nat Methods. 2018;15(8):595–7. 30013044 10.1038/s41592-018-0054-7 PMC6341484

[23] Ebert P, Audano PA, Zhu Q, Rodriguez-Martin B, Porubsky D, Bonder MJ, et al. Haplotype-resolved diverse human genomes and integrated analysis of structural variation. Science. 2021;372(6537):eabf7117. 10.1126/science.abf7117 PMC8026704 33632895

[24] Holt JM, Saunders CT, Rowell WJ, Kronenberg Z, Wenger AM, Eberle M. HiPhase: jointly phasing small, structural, and tandem repeat variants from HiFi sequencing. Bioinformatics. 2024;40(2):btae042. 10.1093/bioinformatics/btae042 PMC10868326 38269623

[25] Marco-Sola S, Moure JC, Moreto M, Espinosa A. Fast gap-affine pairwise alignment using the wavefront algorithm. Bioinformatics. 2021;37(4):456–63. 32915952 10.1093/bioinformatics/btaa777 PMC8355039

[26] Holt J, Saunders C, Dolzhenko E, Krusche P, Olson N, Zook J, et al. Aardvark publication supplementary data bundle Zenodo. 2025. 10.5281/zenodo.17227384.

[27] Holt J, Saunders C, Dolzhenko E, Krusche P, Olson N, Zook J, et al. Aardvark v0.8.0 source code. Zenodo. 2026. 10.5281/zenodo.20271610.

